# Atypical Brain Structures as a Function of Gray Matter Volume (GMV) and Gray Matter Density (GMD) in Young Adults Relating to Autism Spectrum Traits

**DOI:** 10.3389/fpsyg.2020.00523

**Published:** 2020-04-08

**Authors:** Yu Yaxu, Zhiting Ren, Jamie Ward, Qiu Jiang

**Affiliations:** ^1^School of Psychology, Southwest University, Chongqing, China; ^2^Key Laboratory of Cognition and Personality, Ministry of Education, Chongqing, China; ^3^School of Psychology, University of Sussex, Brighton, United Kingdom; ^4^Sackler Centre for Consciousness Science, University of Sussex, Brighton, United Kingdom

**Keywords:** autism spectrum traits, young adults, gray matter volume, gray matter density, voxel-based morphometry

## Abstract

Individuals with autistic traits are those who present in the normal population with characteristics of social, communication, personality, and cognitive impairments but do not meet the clinical threshold for autism spectrum disorder (ASD). Most studies have focused on the abnormalities in ASD patients rather than on individuals with autistic traits. In this study, we focused on the behaviors of a large sample (*N* = 401) of Chinese individuals with different levels of autistic traits, measured using the Autism Spectrum Quotient, and applied voxel-based morphometry (VBM) to determine their association to differences in brain structure. The results mainly showed that the correlation between gray matter volume (GMV) and gray matter density of the brain and the Autism Spectrum Quotient was significant in these regions: the right middle frontal gyrus, which are involved in social processing and social reasoning; the left parahippocampal gyrus, which is involved in socioemotional behaviors and unconscious relational memory encoding; and the right superior parietal lobule, which are involved in cognitive control and the ability to show attention to detail. These findings reveal that people with autistic traits in the normal population have atypical development in GMV and gray matter density, which may affect their social functioning and communication ability.

## Introduction

Autism spectrum disorder (ASD) consists of neurodevelopmental symptoms characterized by core deficits in social functioning but also extending to other cognitive differences (Assaf et al., 2010). The idea of a spectrum captures both the heterogeneity within ASD itself (e.g., from low- to high-functioning intelligence) and the principle of continuity of the symptom profile within the general population itself, in which individuals may exhibit autistic traits to a greater or lesser extent. For instance, the parents of autistic children often report increased levels of autistic traits which are not severe enough to merit a formal diagnosis (Wheelwright et al., 2010). Variation in the level of autistic tendencies is often measured using the Autism Spectrum Quotient (Baron-Cohen et al., 2001). This is a self-report measure that asks about the presence of a range of traits and behaviors commonly seen in autism, including poor social understanding, problems in attention switching, greater attention to detail, poor imagination, and poor communication skills. There has been considerable previous research exploring the brain differences between ASD and controls using functional imaging (Pelphrey et al., 2005; Ikeda et al., 2018) and structural imaging techniques (Critchley et al., 2000; Grelotti et al., 2002; Belmonte et al., 2004; Rojas et al., 2006; Deruelle et al., 2008). However, the study of brain-based individual differences in autistic traits within the general population has received comparatively less attention. This is an important complementary approach that may also have certain advantages. For instance, the overall level of intellectual functioning (a potential confound) is likely to be more homogenous among a student-based neurotypical sample than an ASD sample. The present study examines differences in gray matter linked to autistic traits using a Chinese version of the Autism Spectrum Quotient (AQ) as well as provides important evidence about the neural basis of autistic traits that will potentially contribute to a wider discussion about how autism should be diagnosed and characterized (Pang et al., 2018).

Gray matter volume (GMV) represents the absolute amount of gray matter (Good et al., 2001; Mechelli et al., 2005), whereas gray matter density (GMD) represents the relative concentration of gray matter structures in spatially warped images (i.e., the proportion of gray matter relative to all tissue types within a region) (Mechelli et al., 2005). Focusing on both absolute GMV and GMD may help our understanding of the mechanism of the brain and the individual’s autistic traits.

Previous studies on GMV in ASDs have shown inconsistent results. The existence of studies mostly focused on frontal and temporal regions which are responsible for emotional control and social communication (Hyde et al., 2010; Fan et al., 2012; Morein-Zamir et al., 2016; Patriquin et al., 2016) had long been controversial. First, some studies reported increased GMV in the frontal gyrus (Hyde et al., 2010; Kaiser et al., 2010; Toal et al., 2010; Ecker et al., 2012), while some studies reported decreased GMV in the frontal gyrus (Abell et al., 1999; McAlonan et al., 2005; Takeuchi et al., 2014a; Mori et al., 2015; D’Angelo et al., 2016). Second, some studies reported increased GMV in the temporal gyrus (Abell et al., 1999; Ecker et al., 2012; Lai et al., 2013), while decreased GMV in the temporal gyrus was also reported (Abell et al., 1999; Toal et al., 2010; D’Angelo et al., 2016). There are also studies showing the subcortical brain areas like the basal ganglia extending to the thalamus and the ventral striatum (Takeuchi et al., 2014b). These inconsistent results suggested that the mechanisms of the brain in individuals are still remain unknown. There might exist some other brain regions that closely relate to the autistic traits like the parietal and the parahippocampal regions.

Similar results were obtained in the frontal and the cingulate regions when measuring GMD. A review of a voxel-based morphometry (VBM) study found that GMD decreased in the right paracingulate sulcus and the left inferior frontal gyrus within adults with high-functioning autism (Brambilla, 2003). Another study which explored the relationship between autism and schizophrenia patients within the gray matter and the white matter found that the autism group demonstrated bilateral prefrontal and anterior cingulate increases in contrast with the prefrontal and the left temporal reductions in schizophrenia (Katz et al., 2016). In the three important psychiatric spectra – schizophrenia spectrum disorder, ASD, and obsessive–compulsive disorder – it was found that the GMD of patients did not develop randomly but rather followed identifiable decreased patterns of coalteration in the lateral prefrontal cortex, the ventromedial prefrontal, the orbitofrontal cortex, and the cingulate regions (Cauda et al., 2018). There was also increased GMD shown in the brain regions in autism researches. The neural correlates of executive function in autistic spectrum disorders have shown significant increase in the middle frontal gyrus (MFG) compared with the control groups. Moreover, in individuals with ASD, increased frontal GMD and increased functional activation shared the same anatomical location (Schmitz et al., 2006). Additionally, in a joint behavioral and neuroimaging study of somatosensory and premotor, GMD was significantly higher in the right motor cortex (precentral gyrus) of those with ASD compared to controls (Winter, 2016).

ASD has special characteristics, mainly referring to social deficits, communication disabilities, and repetitive and stereotyped behaviors (Hadjikhani et al., 2005; Troyb et al., 2016). These changes in behavior and mental health are thought to be etiological factors reflected by brain maturation and anatomy (Belmonte et al., 2004; Schmeisser and Boeckers, 2017). While people with autistic traits have not obtained enough attention at a clinical level, they do have an impact on their emotion control, social communication, and interaction (Sucksmith et al., 2011). Individuals with autistic traits undertake the same social responsibilities as normal individuals but psychologically suffer more pain; they are doing the same job as everyone else but taking up more cognitive resources (Sucksmith et al., 2011; Lai et al., 2013). Research into autistic tendencies may result, to some extent, in helping this group ease their burdens and obtain a better, healthier life and therefore preventing a regression to sub-optimal clinical health conditions (Guan and Zhao, 2015). The atypical changes in GMV and GMD, especially in the frontal lobe, the lingual gyrus, the occipital gyrus, the anterior cingulate cortex, the insula, and the parahippocampus during childhood, even to adulthood, within individuals with autistic traits may reflect that these brain regions of the human brain play important roles in cognitive functions, which affect an individual’s brain and behavioral development. As mentioned above, these studies of VBM have been mostly demonstrated on ASD patients but have rarely focused on individuals varying in their levels of autistic traits, especially in the Chinese sample. Therefore, in this study, the participants underwent structural MRI scans after performing an AQ test. The AQ scores were then assessed in relation to GMV and GMD after brain scanning. We hypothesized that the GMV and the GMD of the frontal lobe and the parietal lobe would increase as AQ scores increased and the parahippocampal gyrus (PHG) would decrease as AQ scores increased in individuals with autistic traits since they require more cognitive resources to perform the same work or task as a neurotypical person. This may also be because gray matter maturation is abnormal, while normal gray matter development increases at earlier ages, followed by sustained loss starting around puberty (Gogtay and Thompson, 2010), which may also lead to atypical gray matter development.

## Materials and Methods

### Standard Protocol Approvals, Registration, and Consent

This study and the experimental procedure were approved by the Brain Imaging Center Institutional Review Board of Southwest University of China. In accordance with the Declaration of Helsinki (World Medical Association [WMA], 1991), all participants provided written informed consent and received payment for their time.

### Participants

Four hundred and one individuals (111 men, aged 18–26 years, mean = 21.04 years, standard deviation = 1.27) participated in this research as part of our project investigating associations among genes, brain imaging, and mental health (Liu et al., 2017). Before the experiment, we collected the sample’s basic information to exclude subjects with potential mental disorder; two trained and experienced graduate students in the School of Psychology performed the Structured Clinical Interview for the DMS-IV; all participants included in this study did not meet the DMS-IV criteria for psychiatric disorders and did not use drugs that can affect brain functions. In addition, a self-report checklist was used by all participants to exclude any of following criteria: serious brain trauma, substance abuse, hypertension, or cardiovascular disease. All participants were right-handed, had normal vision, got reasonable payment, and were undergraduates at Southwest University.

### Assessment of AQ

The AQ is a quantitative measure of autistic traits in the general population (Baron-Cohen et al., 2001). The Chinese version of AQ (Lau et al., 2013) was used in this study which consists of the social skill, communication, attention switching, imagination, and attention to detail subscales contained within 50 statements, to which the participants responded on a four-point Likert scale: “definitely agree” or “slightly agree” responses scored one point, while “slightly disagree” or “definitely disagree” responses scored one point in reverse options. In half of the statements, the diagnostic answer is “agree,” and in the other half “disagree.” One point is awarded for each diagnostic answer which results in a continuous distribution of scores in the population sample. The total score ranges from 0 to 50 points, with higher scores suggesting a greater magnitude of autistic traits. Currently available data from research on the properties of this scale indicate that the measurement reliability for the total score is satisfactory (Austin, 2005; Hurst et al., 2007; Hoekstra et al., 2008; Ingersoll et al., 2011; Kloosterman et al., 2011). In the present study, we focused on analyzing the total AQ score.

### Image Acquisition

A 3-T Siemens Trio MRI scanner (Siemens Medical, Erlangen, Germany) was used to gather images. Then, high-resolution T1-weighted structural images (repetition time = 1,900 ms, inversion time = 900 ms, flip angle = 9°, echo time = 2.52 ms, 256 × 256 matrix, 176 slices, 1.0 mm slice thickness, and voxel size = 1 × 1 × 1 mm^3^) were collected, on which a magnetization-prepared rapid gradient echo (MPRAGE) sequence was used.

### MRI Preprocessing

The structural magnetic resonance (MR) images were processed with SPM8^1^ implemented in MATLAB R2014a (MathWorks Inc., Natick, MA, United States). First, every magnetic resonance image was displayed in SPM8 to monitor artifacts and obvious anatomical abnormalities. Then, VBM was performed with diffeomorphic anatomical registration using exponentiated lie algebra (DARTEL) (Ashburner, 2007). The new segment toolbox from SPM8 was applied to every T1-weighted MR image to extract tissue maps corresponding to gray matter, white matter, and cerebrospinal fluid in the native space. The DARTEL template creation toolbox was used to improve intersubject alignment. The resliced images of the gray and white matter were registered to a subject-specific template, and subsequently the normalization function in the DARTEL toolbox was used to normalize the individual images of gray and white matter to the MNI space (1.5 mm isotropic voxels). Finally, each subject’s gray and white matter maps were warped using their corresponding smoothed (10-mm full-width at half-maximum Gaussian kernel) and reversible deformation parameters to the custom template space and then to the MNI standard space. GMV images were modulated by calculating the Jacobian determinants derived from the special normalization step and by multiplying each voxel by the relative change in volume.

### Statistical Analysis

We applied multiple linear regression to identify the brain regions whose GMV and GMD were associated with individual differences in the AQ within SPM8. In this study, a customized binary mask was used to avoid the partial volume effect by including voxels with a gray matter value >0.2. All subsequent statistical analyses were conducted in this mask. To remove potential confounds, we used age and total GMV and GMD mean values as nuisance covariates. Clusters with continuous suprathreshold voxels (*p* < 0.001) were initially identified within the custom mask and within the AlphaSim correction for multiple-comparison in DPABI. Many studies used smoothness kernel in preprocessing to estimate the biggest cluster size with AlphaSim correction; however, the effective smoothness is bigger than the applied because the pre-smoothed image has implicit smoothness. Simply inputting Gaussian smoothing kernel that was applied during preprocessing to AlphaSim is incorrect (Bennett et al., 2009). DPABI prevents that kind of errors by performing AlphaSim correction based on the estimated effective smoothness (Yang et al., 2016). The minimum cluster size in AlphaSim correction of GMV-positive and GMV-negative was *k* >192. The minimum cluster size in AlphaSim correction of GMD positive was *k* >50, respectively. The p-maps were thresholded to yield an expected *p*-value of <0.05, voxel-wise *p* < 0.005.

## Results

### Descriptive Statistics

The demographic data and behavioral results are shown in Table 1. The mean AQ score of the current sample was 19.58, and the standard deviation was 5.27. The difference in AQ between gender are shown in Supplementary Table S1.

**TABLE 1:** A summary of the demographic information in the present study.

Measure	*N* = 401		
	Mean	*SD*	Range
Age	21.04	1.27	18–26
AQ (total)	19.58	5.72	7–37
Social skill	3.75	2.47	0–10
Attention switching	5.14	1.63	1–10
Attention to detail	4.66	2.13	0–10
Communication	3.09	1.93	0–10
Imagination	2.94	1.62	0–7

*AQ, Autism Spectrum Quotient; SD, standard deviation; N, number.*

### Correlations Between GMV and AQ Score

**FIGURE 1:**
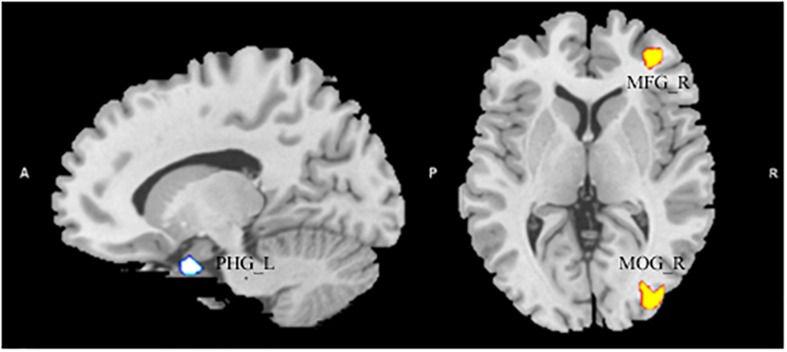
The significant positive correlation between gray matter volume (GMV) and Autism Spectrum Quotient score showing areas in the right middle frontal gyrus and in the middle occipital gyrus, and had a significant negative association with the GMV in the left parahippocampal gyrus. Alphasim-corrected cluster level *p* < 0.05, voxel-wise level *p* < 0.005.

After entering age, gender, and global volumes of gray matter as covariates into the regression model, a multiple regression analysis revealed that the AQ (total) score had a significant positive association with the GMV in the MFG and the middle occipital gyrus (MNI coordinates: 10, 33, 1.5, *T* = 3.90; 30, -88.5, 0, *T* = 3.22). Additionally, the AQ score had a significant negative association with the GMV in the left parahippocampal gyrus (MNI coordinates: -13.5, 3, -27, *T* = -3.79) (see Figure 1 and Table 2).

**TABLE 2:** The brain regions in gray matter volume significantly correlated with Autism Spectrum Quotient.

	Item	Voxels	H	T	BA	MNI			Brain regions
Positive	AQ	195	R	3.90	10	33	48	1.5	Middle frontal gyrus
		313	R	3.22	18	30	−88.5	0	Middle occipital gyrus
Negative	AQ	118	L	−3.79	n/a	−13.5	3	−27	Parahippocampal gyrus

*MNI, Montreal Neurological Institute; H, hemisphere; L, left; BA, Brodmann areas; n/a, not available. Alphasim-corrected cluster level p < 0.05, voxel-wise level p < 0.005.*

### Correlations Between GMD and AQ Score

**FIGURE 2:**
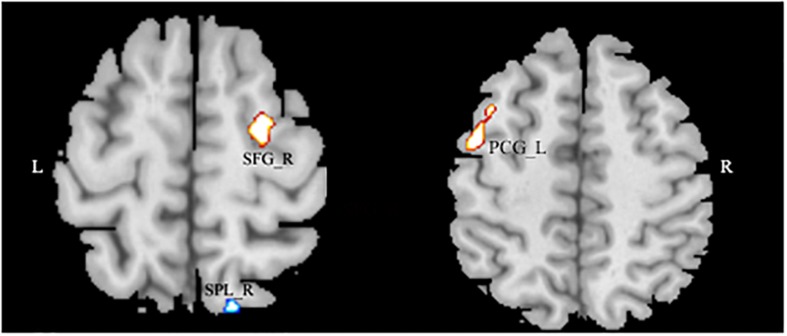
The significant negative correlation between gray matter volume and Autism Spectrum Quotient score showing areas in the left superior frontal gyrus and in the right precentral gyrus, and negative in the right superior parietal lobule. Alphasim-corrected cluster level *p* < 0.05, voxel-wise level *p* < 0.005.

After entering the age, the gender, and the global density of gray matter as covariates into the regression model, a multiple regression analysis revealed that the AQ (total) score had a significant positive association with the GMD in the left superior frontal gyrus (SFG), right precentral gyrus, and negative in the right superior parietal lobule (SPL) (MNI coordinates: 25.5, -6, 63, *T* = 3.60; -45, -1.5, 52.5, *T* = 3.33; 13.5, -73.5, 58.5; *T* = -3.18) (see Figure 2 and Table 3).

**TABLE 3:** The positive correlation between gray matter density and Autism Spectrum Quotient.

	Item	voxels	H	T	BA	MNI			Brain regions
Positive	AQ	124	L	3.60	10	25.5	−6	63	Superior frontal gyrus
		108	R	3.33	24	−45	−1.5	52.5	Precentral gyrus
Negative	AQ	50	R	−3.18	7	13.5	−73.5	58.5	Superior parietal lobule

*MNI, Montreal Neurological Institute; H, hemisphere; L, left; BA, Brodmann areas; n/a, not available. Alphasim-corrected cluster level p < 0.05, voxel-wise p < 0.005.*

We also explore the associations between the sub-dimensions of AQ and the brain areas of GMV and GMD and found significant associations in social skill, attention switching, and communication parts (see Supplementary Table S2).

## Discussion

In this study, we investigated associations between the GMV and the GMD of brain structures and the AQ of individuals with autistic traits. Our VBM analysis results showed that the AQ (total) score had a significant positive association with GMV in the right MFG and the right middle occipital gyrus and a negative association to left PHG. The AQ (total) score had a significant positive correlation with the GMD in the left SFG and the right precentral gyrus and a negative association with the right SPL and the right MFG. These findings point to disturbances of brain growth and maturation as an important pathomechanism. The atypical development in GMV and GMD in the brain structures of individuals with autistic traits may explain the abnormal neuro and social behaviors to some degree.

In the present study, we focused on the brain regions that are closely related to the autistic traits. First, the AQ (total) score was significantly and positively correlated with gray matter in an extensive region that included the right MFG and SFG of gray matter, which is in line with a previous study and involved in social communication and interaction (Hyde et al., 2010; Fan et al., 2012; Marsh et al., 2014; Morein-Zamir et al., 2016; Patriquin et al., 2016; Stevenson et al., 2018); these two regions are also known to be involved in planning, flexibility, executive functioning, and working memory in ASD (Zelazo and Müller, 2002; Hill, 2004; Jurado and Rosselli, 2007; Craig et al., 2016). These findings collectively suggested atypical development in the structure and functions of the brain; indeed neuropsychological and neuroimaging studies performed thus far have suggested the association between people with autistic traits and increases in MFG and SFG volumes which may be influenced by executive dysfunction and social communication deficits (Booth et al., 2003; Corbett et al., 2009; Vanegas and Davidson, 2015; Craig et al., 2016). People with autistic traits who have normal intelligence, normal lifestyle, and seemingly normal social interactions are burdened with more social pressure, which may be due to their social executive dysfunction (Bayliss and Tipper, 2005; Christ et al., 2010). The discrepancy between social communication situational pressures and actual social abilities is consequent to the compensatory strategies of adults with autistic traits or of clinical ASD patients that bring task performance to ceiling levels (Schneider et al., 2013, 2014). The atypical development in the MFG and SFG could reflect a lack of pruning during the normal growth spurt, leading to excessive preservation of unneeded increases. Such an effect would certainly lead to abnormal structure between individuals with autistic traits and brain regions.

Second, the significant decrease in the left PHG of GMV was consistent with previous neuroimaging findings in adults and children with autism (Page et al., 2009; Kosaka et al., 2010; Mueller et al., 2013; Yu et al., 2019), Moreover, severely restricted and repetitive behaviors were associated with the PHG (Monk et al., 2009; Weng et al., 2010; Hau et al., 2019). These two brain regions are implicated in unconscious relational memory encoding, autobiographical memory (Tanweer et al., 2010; Duss et al., 2014), and socioemotional behaviors (Dawson, 1991; Yang et al., 2016; Puiu et al., 2018) that are abnormal in individuals with autistic traits and in patients with clinical ASD, such as understanding the mental state of others, emotion processing, and language (Barrett et al., 2007; Lartseva et al., 2015; Grecucci et al., 2016). As previously reported, individuals with autistic traits could communicate with others in a normal way but are burdened with more social pressure (El Kaliouby et al., 2006). Individuals with autistic traits show deficits in social communication or social avoidance and do not have compensatory strategies, which influence the relationship between cognitive functions and neural system (Senju et al., 2011; Low and Watts, 2013). Additionally, individuals with autistic traits usually remembered more adverse events like social rejection, childhood trauma, and daily interpersonal stress (Sebastian, 2015). Young adults have the ability to integrate emotional information into a proper degree that need wholesome neurological development (Sebastian, 2015). Neuroimaging evidence is now showing that improvements in social cognition during young adults are underpinned by the ongoing development in relevant regions (Blakemore, 2008). This may be the reason for the decrease in GMV of the left PHG as reflected in the current study and may explain atypical behaviors such as poor communication disorder and repetitive behaviors within individuals with autistic traits.

Third, we also found that individuals with autistic traits had a decrease in GMD in the right SPL which was inconsistent with previous study, which might be the case given that the large sample has more weight in this study than others. The SPL is important in cognitive control and attention to detail (Koechlin and Hyafil, 2007; Kompus et al., 2009; Qiu et al., 2018). To identity brain regions that differed in activity during social and non-social orienting, Greene’s study found that the ASD group demonstrated significant activation in the SPL (Greene et al., 2011). In a facial processing research, improved facial affected recognition performance which was accompanied by higher activation of the right SPL (Bolte et al., 2006), which indicated that the decrease of SPL in the present study within individuals with autistic traits might influence social cognition deficits. The SPL also composed the frontal–parietal control (FPC) network (Koechlin et al., 1999; Kompus et al., 2009; Mundy, 2018), and deficits to this phenomenon in brain regions may explain the non-social difficulties in individuals with autistic traits, such as repetitive, poorly controlled, and poor goal-directed action (Takarae et al., 2014; de Wit, 2018). The FPC system has been identified as supporting cognitive control and decision-making processing (Vincent et al., 2008). Relative to typical individuals, the social failure of individuals with autistic traits to process information globally might be argued to follow from problems in shifting between local and global processing (Bogousslavsky et al., 1987; Frith, 2004; Liss et al., 2008; Van Eylen et al., 2018), and a failure of cognitive control may be the neural basis of the autistic traits in these individuals (Vartanian et al., 2018). These atypical social cognitive functions relative to SPL was found within individuals with autistic traits in this study, indicating that social cognition deficit not only influences people’s behavior strongly but also results in a unique neuroanatomical structure. Above all, these atypical developments in brain structures, such as the MFG SFG, the left PHG, and the SPL, may play a role in social and attention abilities associated with brain function.

## Conclusion

In summary, the present study replicated a previous study and broadened our understanding of the neural mechanisms underlying autistic traits within young adults. We found that the MFG, the SFG, the PHG, and the SPL brain regions play an important role in individuals with autistic traits; these brain regions are involved in some cognitive function deficits like social communication, cognitive control, attention to detail, and socioemotional behaviors. These abnormalities are consistent in young adults with autistic traits, which are reflected in specific brain regions. The current study that interpret individuals with autistic traits can help young people know themselves and integrate into their social life better in some degree.

## Limitations

The current study investigates the brain areas of GMV and GMD in a large sample of young adults with autistic traits. The results showed that social communication, cognitive control, and some other brain functions are linked to brain areas in GMV and GMD, which replicated a previous study and broadened our understanding of the neural mechanism underlying autistic traits. Meanwhile, there are also deficiencies in the following three aspects: first, our autistic traits sample are all university students; this may have a bias in it compared to average level. Second, in our sample, there is a difference between genders. In a future study, we will further explore this difference using a larger sample wherein there is balancing of gender difference between men and women. Third, we want to further explore the neural mechanism using more different brain types, like resting-state functional connectivity and task-based functional connectivity, among others.

## Data Availability Statement

The datasets generated for this study are available on request to the corresponding author.

## Ethics Statement

The studies involving human participants were reviewed and approved by Brain Imaging Center Institutional Review Board of Southwest University of China. The patients/participants provided their written informed consent to participate in this study. Written informed consent was obtained from the individual(s) for the publication of any potentially identifiable images or data included in this article.

## Author Contributions

QJ conceived the experiments. YY and ZR conducted the experiments, analyzed the results, and carried on manuscript writing. JW provided the AQ questionnaire and proposed many constructive advices on the manuscript. All authors reviewed the manuscript.

## Conflict of Interest

The authors declare that the research was conducted in the absence of any commercial or financial relationships that could be construed as a potential conflict of interest.

## Footnotes

1 https://www.fil.ion.ucl.ac.uk/spm/
