# Supplementary material for: Atypical Brain Structures as a Function of Gray Matter Volume (GMV) and Gray Matter Density (GMD) in Young Adults Relating to Autism Spectrum Traits
Source: Front Psychol. 2020 Apr 8;11:523. doi: 10.3389/fpsyg.2020.00523 (PMC7158890; doi:10.3389/fpsyg.2020.00523)
Supplement: Supplementary file 1 [file Table_1.docx]

**Supplementary Table S1. The gender difference among AQ (total) and sub-dimension**

|  | **Men (n = 111)** | | **Woman (n = 290)** | |  |
| --- | --- | --- | --- | --- | --- |
| **Items** | **Mean** | **SD** | **Mean** | **SD** | **T** |
| AQ (total) | 20.72 | 5.33 | 19.14 | 5.19 | 2.72****** |
| Social Skill | 4.10 | 2.44 | 3.61 | 2.48 | 1.77 |
| Attention Switching | 5.01 | 1.57 | 5.18 | 1.66 | -0.92 |
| Attention to detail | 4.73 | 2.01 | 4.64 | 2.17 | 0.39 |
| Communication | 3.44 | 1.98 | 2.96 | 1.90 | 2.26***** |
| Imagination | 3.44 | 179 | 2.75 | 1.52 | 3.86****** |

*Notes: * p < 0.05; ** p < 0.01; SD: standard deviation*
