# Supplementary material for: Atypical Brain Structures as a Function of Gray Matter Volume (GMV) and Gray Matter Density (GMD) in Young Adults Relating to Autism Spectrum Traits
Source: Front Psychol. 2020 Apr 8;11:523. doi: 10.3389/fpsyg.2020.00523 (PMC7158890; doi:10.3389/fpsyg.2020.00523)
Supplement: Supplementary file 2 [file Table_2.docx]

**Supplementary Table S2. The correlation results of sub-dimension value and brain areas**

| Items | MFG_GMV | SFG_GMD | SPL_GMD |
| --- | --- | --- | --- |
| AQ total  Social Skill  Attention Switching  Attention to Detail  Communication  Imagination | **.047*** | **.008**** | **002**** |
|  | .220 | **.014*** | **.003**** |
|  | **.036*** | **.011*** | **.000**** |
|  | **.005**** | .168 | .083 |
|  | **.027*** | **.000**** | .108 |
|  | .365 | .169 | .399 |

*Notes: * p < 0.05; ** p < 0.01.*

*MFG: middle frontal gyrus; SPL: superior parietal gyrus; GMV: gray matter volume; GMD: gray matter density*
